# Rare Earth Elements in Heat-Resistant Magnesium Alloys: Mechanisms, Performance, and Design Strategies

**DOI:** 10.3390/ma18174090

**Published:** 2025-09-01

**Authors:** Zheng Tian, Chong Wang, Bai-Xin Dong, Hong-Yu Yang, Lei Zhan, Dan Luo, Feng Qiu, Qi-Chuan Jiang

**Affiliations:** 1 School of Engineering, Changchun Normal University, Changchun 130032, China; tianzheng@ccsfu.edu.cn (Z.T.);; 2 Key Laboratory of Automobile Materials, Ministry of Education, Department of Materials Science and Engineering, Jilin University, Renmin Street No. 5988, Changchun 130025, China; 3 School of Automotive Engineering, Jilin Communications Polytechnic, Changchun 130012, China; 4 School of Materials Science and Engineering, Wuhan University of Technology, Wuhan 430070, China

**Keywords:** magnesium alloys, rare earth, heat-resistant, mechanical properties, strengthening mechanisms

## Abstract

This study investigates the influence of RE elements on the room- and high-temperature properties of magnesium alloys. The effects of RE type, addition level, and multi-element alloying strategies were systematically analyzed to clarify the underlying strengthening mechanisms and processing pathways for optimizing Mg–RE alloys. RE elements enhance the mechanical and thermal properties of Mg alloys through crystal structure modification, formation of thermally stable dispersed phases, precipitation strengthening, and solid-solution strengthening. Compared with conventional alloying elements, RE additions offer distinct advantages in strengthening efficiency and overall performance. To fully exploit these benefits, new research paradigms that integrate machine learning and other advanced techniques are required, enabling the intelligent design of multicomponent alloy systems tailored to specific application requirements.

## 1. Introduction

Magnesium alloys are widely used due to their lightweight characteristics, particularly in aviation and transportation systems, often as components of power systems or conformal shells (e.g., Me109 fighter, 1935; Volkswagen Beetle, 1936). Their working environment requires resistance to the heat dissipation temperature of the power system, usually around 100 °C. However, with the development of power systems in transportation vehicles, higher power output and more confined internal spaces often result in higher environmental temperatures. When the operating temperature exceeds 150 °C, magnesium alloys are prone to creep and oxidation, leading to significant deterioration of their mechanical properties. This challenge to the high-temperature performance of magnesium alloys limits the application of commercial magnesium alloys. To enhance the high-temperature resistance of alloys, silver (Ag) was initially incorporated into magnesium alloys, resulting in the formation of Mg-Ag alloys, which were subsequently employed in the transmission systems of Sikorsky helicopters. However, due to the high cost of silver, it was replaced by rare earth (RE) elements in Mg-Ag alloys, leading to the development of novel Mg–RE alloys that effectively improve the high-temperature performance of the alloys.

RE elements can substantially enhance the mechanical and thermal properties of magnesium alloys through mechanisms such as crystal structure optimization, formation of thermally stable dispersed phases, precipitation strengthening, and solid solution strengthening. Research findings [1,2,3,4,5,6] have demonstrated that, whether as primary alloying elements or as modifiers, RE additions to magnesium alloys can significantly improve their heat resistance, elevating the service temperature from around 100 °C to as high as 300 °C. This performance is markedly superior to that of conventional commercial magnesium alloys and even many aluminum alloys.

Alloy systems such as Mg-RE-Zr and Mg-Al-RE, developed through the addition of RE elements, have shown significant improvements in thermal resistance and excellent mechanical performance at both room and elevated temperatures [1,2,3,4,5,6,7,8,9,10,11,12]. Since the 1960s, RE elements have been recognized as the most effective alloying elements for improving the high-temperature performance of magnesium alloys. RE-containing magnesium alloys are also the most widely used magnesium alloys in high-temperature applications. For example, WE-series magnesium alloys are extensively utilized in aerospace and defense for components that function within the temperature range of 200–300 °C, while AE-series magnesium alloys are used in transportation for components such as automotive engine mounts operating at 150–200 °C. Nevertheless, the development of these alloy systems remains incomplete. Excessively high RE contents may lead to grain boundary coarsening, increased production costs, and deteriorated workability. Although RE elements can be added to magnesium alloys in relatively high proportions and even serve some functions such as hydrogen storage, their systematic addition levels are typically constrained by several factors when magnesium alloys are employed in structural components: Higher RE content increases brittleness, limiting application scenarios; The strengthening effect of RE additions has a certain threshold, beyond which further increases do not yield additional property improvements; The limited reserves and exorbitant costs of REs significantly impede their large-scale application.

For example, WE-series magnesium alloys contain up to 12% RE but are generally limited to high-value applications such as aerospace, defense, and medical fields. In contrast, Mg-Al-RE alloys typically contain less than 2% RE, with costs affordable for applications in automobiles and electrical equipment. In summary, the RE content in magnesium alloys greatly depends on their cost and processing challenges, and has always been constrained by practical application demands.

Therefore, to achieve a balance among cost, room-temperature performance, and high-temperature performance, and to facilitate broader applications of magnesium alloys, it is imperative to develop an in-depth understanding of the strengthening mechanisms of RE elements in magnesium alloys and to optimize alloy compositions. Against this backdrop, the present article provides a comprehensive overview of the mechanical properties, heat resistance, and strengthening mechanisms of RE-doped magnesium alloys. Furthermore, it discusses current challenges and offers insights into potential future research directions aimed at developing magnesium alloys with superior high-temperature performance and optimized comprehensive properties.

## 2. Mechanics and Heat-Resistance Strengthening Mechanism

### 2.1. Strengthening Mechanisms of Mechanical Properties

In magnesium alloys, RE elements contribute to mechanical strengthening through multiple mechanisms, primarily including solid solution strengthening, precipitation strengthening, and fine grain strengthening.

Grain refinement induced by RE elements occurs throughout the entire alloy preparation process. During solidification, RE-containing phases can function as heterogeneous nucleation sites, attributed to the interfacial energy relationship between the α-Mg matrix and the RE phase. Experimental investigations [13,14,15] have demonstrated that when RE phases serve as such nucleation sites, they can effectively reduce the as-cast grain size from 200–500 μm to 20–50 μm. The resulting Hall–Petch effect enhances room-temperature yield strength by approximately 80–120 MPa. This refinement effect is rooted in the lattice compatibility between RE elements and the Mg matrix. For example [1,5], Gd, with its face-centered cubic structure, forms a semi-coherent interface with the basal plane of α-Mg, providing superior nucleation potency compared with conventional Al-Mn intermetallic compounds.

During thermomechanical processing, RE elements also exert a significant influence on dynamic recrystallization (DRX), exhibiting distinct temperature sensitivity. The DRX behavior during extrusion is strongly affected by RE additions. For instance [5], adding 1.5% Y markedly reduces the activation energy of recrystallization, thereby promoting the formation of small and dispersed equiaxed grains. This is attributed to the dual role of Y in dislocation dynamics: (1) Y atoms tend to segregate around dislocation lines to form Cottrell atmospheres, reducing dislocation mobility; and (2) Y facilitates the formation of sub-grain boundaries during dynamic recovery, providing nucleation sites for recrystallization. An optimal balance of these competing effects is typically achieved within 300–400 °C, resulting in uniform equiaxed grains of 5–8 μm and significant improvements in mechanical properties. It is noteworthy that different RE elements induce distinct recrystallization behaviors: heavy REs (e.g., Yb) tend to promote continuous DRX, whereas light REs (e.g., Ce) are more prone to discontinuous DRX. This grain refinement effect not only enhances yield strength at room temperature by 80–120 MPa but also improves creep resistance at elevated temperatures.

Solid solution strengthening and precipitation strengthening represent two additional principal mechanisms by which RE elements improve mechanical performance. These effects arise from three synergistic contributions: (1) lattice distortion caused by solute atoms; (2) the impediment of dislocation motion via the Orowan mechanism by precipitates; and (3) grain boundary pinning by thermally stable phases. The solid solubility of RE elements in the Mg matrix is highly temperature dependent. For Gd element [3,7], its solubility can reach approximately 4.5 wt.% at 500 °C, but decrease to 0.8 wt.% at room temperature, enabling precipitation hardening through appropriate heat treatments. Aging treatments such as T6 can induce the precipitation of γ″ (Mg_5_RE) and β′ (Mg_7_RE) phases, forming a multi-scale precipitate architecture. These nanoscale precipitates effectively obstruct dislocation motion via the Orowan mechanism, enabling alloys such as WE43 to achieve yield strength as high as 250 MPa [16,17].

**Figure 1:**
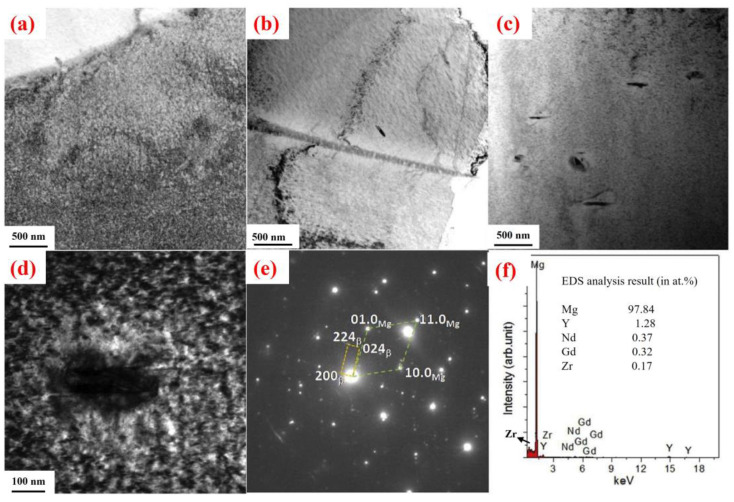
The microstructures of Mg-5Y-2.5Nd-1.5Gd-0.5Zr alloy peak-aged at (**a**) 200 °C, (**b**) 225 °C, and (**c**) 250 °C; (**d**) BF-TEM image; (**e**) the corresponding SAED pattern; (**f**) the point EDS spectrum and analysis result for the large precipitate [13].

The thermal stability of RE-containing precipitates also plays a crucial role in determining alloy performance. In the Mg–5Y–2.5Nd–1.5Gd–0.5Zr alloy, the size and distribution of RE precipitates exhibit notable temperature dependence [13]. As illustrated in Figure 1, precipitate size remains nearly unchanged at 200 °C; slight coarsening is observed near grain boundaries at 225 °C, but the variations remain relatively small, with significant growth occurring only at 250 °C. This thermal stability of RE precipitates directly contributes to the high-temperature mechanical performance of the alloy.

### 2.2. Optimization of Thermal Resistance

RE elements, owing to their large atomic radii and high chemical reactivity, play a pivotal role in enhancing the thermal resistance of magnesium alloys. These elements readily form thermally stable compounds with the Mg matrix, including high-melting-point intermetallics such as Al_11_Ce_3_ (melting point > 1000 °C) and nanoscale precipitates. The formation of these multi-scale strengthening structures not only provides precipitation hardening but also ensures exceptional thermal stability at elevated temperatures. This stability effectively suppresses grain boundary sliding, thereby improving the high-temperature performance of the alloy. RE-induced thermal enhancement in magnesium alloys primarily occurs through three mechanisms: grain boundary strengthening, stabilization of precipitates, and surface protection. At the grain boundary scale, the segregation of RE atoms induces nanoscale atomic clusters, which act as potent pinning centers to impede grain boundary sliding at elevated temperatures. Experimental studies [18,19,20,21,22] have shown that the addition of 2.5–4.0 wt.% RE elements (e.g., Gd or Y) can elevate the maximum service temperature of magnesium alloys from 120 °C to 300 °C, with tensile strengths at 200 °C retaining up to 80% of their room-temperature values. For instance, Mg–10Gd–3Y–0.5Zr as-cast alloys exhibit yield strengths up to 220 MPa at 250 °C [23,24].

At the precipitate level, the uniform dispersion of high-melting-point intermetallic compounds forms a three-dimensional reinforcing network. Empirical data [19,20,21,22] indicate that when RE content is 2.5–4.0 wt.%, the interparticle spacing of precipitates decreases from 1.2 μm to 0.3 μm, thereby increasing tensile strength retention at 200 °C from 60% to 80%.

Because the morphology and distribution of precipitates directly affect the tensile and creep properties of magnesium alloys at high temperatures, RE content is generally positively correlated with thermal performance. As summarized in Table 1, although Al–RE intermetallic phases exhibit high melting points, their strengthening contribution is limited at low RE contents, in contrast to the more thermally stable phases in Mg–RE alloys. For instance, Mg–Zn–RE alloys are limited to ~200 °C due to the inadequate stability of their primary MgZnRE strengthening phase. In contrast, Mg–Gd–Y alloys, which precipitate thermally stable β′ phases, can sustain performance up to 300 °C. However, when RE content exceeds 5 wt.%, coarse primary phases form more readily. After exposure at 200 °C for 50 h, the average precipitate size increases from 80 nm to 220 nm, significantly reducing creep resistance [25].

**Figure 2:**
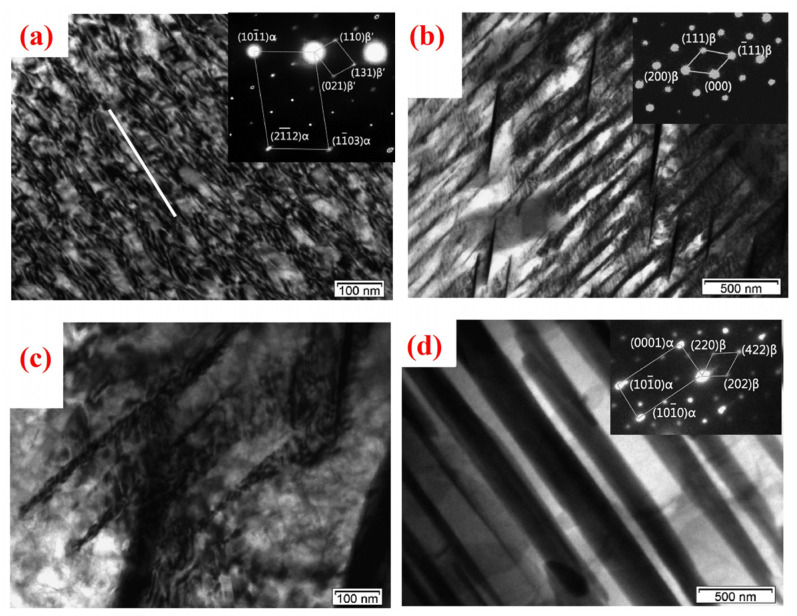
TEM images of Mg-10Gd-3Y-2Nd-0.5Zr alloy after creep for 100 h under different temperatures and loads: (**a**) 200 °C/70 MPa, (**b**,**c**) 250 °C/70 MPa, (**d**) 300 °C/70 MPa [26].

The morphology and thermal evolution of precipitates critically influence high-temperature behavior. For example, in Mg–10Gd–3Y–2Nd–0.5Zr alloy [26], as shown in Figure 2, after 100 h of creep under 70 MPa at 200–300 °C, the precipitates at 200 °C are fine, dense β′ phases with similar orientation. At 250 °C, the precipitates coarsen into β phases of 0.5–2 μm, distributed in needle- or lath-like morphologies at ~45°, and exhibit strong dislocation entanglement. These interlocking β phases effectively impede dislocation motion, enhancing creep resistance. However, at 300 °C, significant coarsening of the β phase occurs. These coarse precipitates, enriched in RE, reduce the available RE atoms in solid solution, weakening solid-solution strengthening in the α-Mg matrix. Moreover, increased interparticle spacing facilitates dislocation motion, accelerating creep deformation. Thus, precipitate morphology and thermal evolution are interrelated and constitute key determinants of high-temperature alloy performance.

In addition, RE elements improve oxidation resistance by modifying the composition and structure of surface oxide films. Dense RE_2_O_3_ layers reduce the oxidation rate at 300 °C by 40–70% compared to conventional magnesium alloys [1,3]. However, excessive RE content can cause microstructural coarsening and increased defect density, ultimately degrading alloy performance [1,2,3,4,5,6,7,25,27]. Therefore, a balance must be achieved between precipitate density and thermal stability, suggesting an optimal RE content range. Related research [23,24,25,26,27,28,29,30,31] indicated that the optimum performance occurs at 2–5 wt.% RE. Excessive additions (>7 wt.%) lead to precipitate coarsening (>500 nm) and increased interfacial defect density, reducing ductility.

Heat treatment parameters can also strongly impact the thermal performance of Mg-RE alloys. For the Mg–Gd–Y–Zr alloy [23,24], the increase in the solution temperature from 500 °C to 540 °C can readily raise the Gd solubility from 3.2 to 4.1 wt.%, enhancing precipitate density by 40% after aging and increasing yield strength at 250 °C from 180 MPa to 220 MPa. However, excessive solution temperatures (>550 °C) cause abnormal grain growth and degrade mechanical properties. This nonlinear relationship requires precise control of the heat treatment window. A two-stage aging process (e.g., 180 °C for 24 h followed by 250 °C for 8 h) is typically employed to optimize precipitate size distribution and balance strengthening mechanisms.

### 2.3. The Effect of Multi-Element Alloying

Studies have demonstrated [1,2,3,4,5,6,27,28,29,30,31,32,33] that the co-addition of different RE elements can produce unique synergistic effects. The combined incorporation of multiple RE elements reduces their individual solubilities, thereby significantly increasing the precipitate density without raising the total RE content. This approach not only enhances the strengthening effect but also mitigates the detrimental impacts associated with excessive RE additions, such as reduced ductility and poorer workability. Consequently, optimizing the combination of RE elements more effectively improves the precipitation efficiency of RE additions, providing a practical strategy to enhance the overall performance of magnesium alloys without increasing RE content.

The addition of 0.3–0.5 wt.% Zn has been found to promote the formation of long-period stacking ordered (LPSO) structures, which synergistically enhance both strength and toughness of the alloy [4,19,22]. Ternary RE combinations exhibit superior high-temperature properties. For instance, compared with the addition of a single RE element, the incorporation of a Gd/Y/Nd ternary RE mixtures with a total content of 4.5 wt.% [34,35] extends the creep life of the alloy at 300 °C by 3–5 times. This improvement is mainly attributed to the multi-scale synergistic strengthening contributions from β′ and γ″ phases and LPSO structures: the β′ phase impedes dislocation motion, the γ″ phase suppresses grain boundary migration, and the LPSO structure enhances interfacial stability, delaying crack initiation.

In the Mg–Gd–Y–Zr system, the contents of Gd and Y are critical determinants of mechanical strength. When the atomic ratio of Gd to Y reaches 1.5–2.0, the coherency of the β′ phase is optimized, and the lattice mismatch decreases from 3.2% to 1.8% [1,34]. This structural advantage raises the energy barrier for dislocation bypass, altering the deformation mechanism. Specifically, whereas dislocations in single RE-containing alloys typically bypass precipitates via Orowan looping, in dual-RE systems, the prevalence of dislocation cross-slip is significantly enhanced. This transition in deformation mode facilitates concurrent improvement of both strength and ductility.

**Figure 3:**
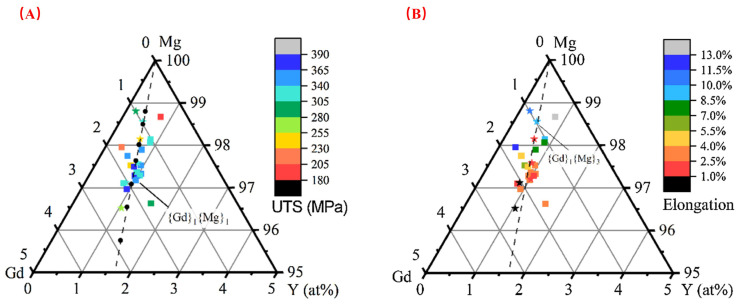
Correlation between mechanical properties (in color depth) and Mg-Gd-Y alloy compositions: (**A**) tensile strength, (**B**) elongation [34].

Currently, most high-strength Mg–Gd–Y–Zr alloys fall within a narrow compositional range. As shown in Figure 3, there is a clear relationship between mechanical properties and the content of Mg, Gd, and Y. In Figure 3A, for a constant Gd content, an elevation in Y content leads to an increase in the ultimate tensile strength (UTS). Similarly, with a fixed Y content, a rise in Gd concentration also results in an enhancement of UTS. The UTS reaches a maximum when the atomic ratio of Gd to Y approaches 2:1. However, as shown in Figure 3B, elongation exhibits the opposite trend, decreasing as strength increases.

Nevertheless, the negative effects of excessive hybridization must be carefully considered. When the number of RE elements exceeds three, the resulting complexity in phase composition and disparities in driving forces for phase transformations can disrupt the precipitation sequence [36]. This often leads to coarsening of precipitates and a reduction in strengthening efficiency. Furthermore, interactions between RE elements and other alloying components must be precisely controlled. For example, in the Mg–Al system, Y contents exceeding 0.2 wt.% promote the formation of Al_2_Y intermetallic compounds, which increase melt viscosity and can cause casting defects [37,38].

### 2.4. Optimization of Alloy Composition

To achieve precise control over the properties of multicomponent magnesium alloys, systematic optimization must be conducted in three aspects: atomic-scale structural features, thermodynamic stability, and electronic structure. Among these, atomic radius matching between alloying elements is a primary design principle [39,40,41]. RE elements, which typically have larger atomic radii, induce significant lattice distortion when dissolved in the magnesium matrix, leading to lattice expansion. While such distortion is beneficial for solid-solution strengthening, excessive atomic size mismatch can promote the formation of unstable or brittle precipitates, severely compromising mechanical properties.

**Figure 4:**
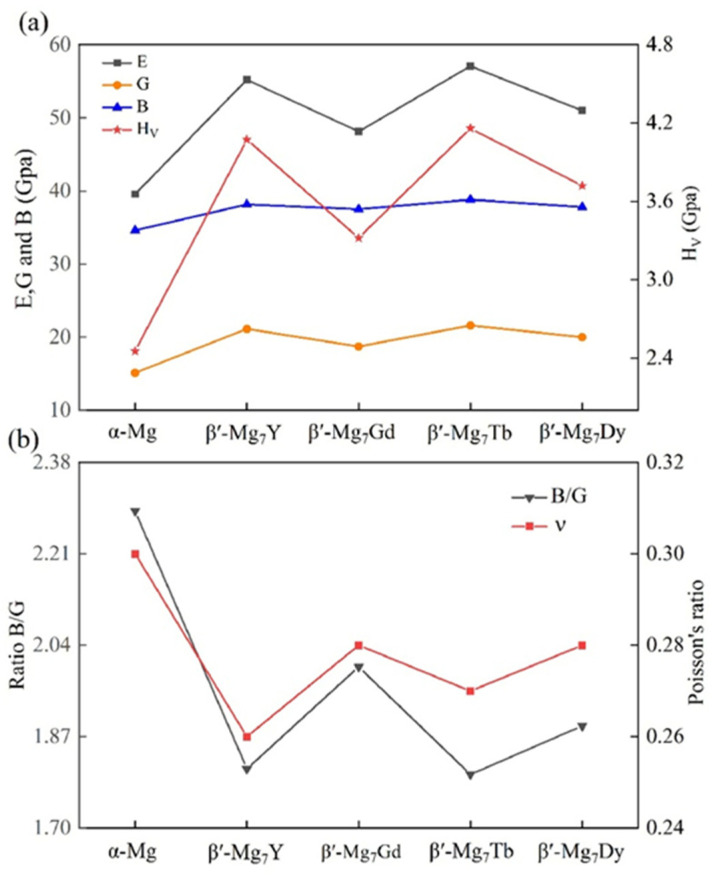
(**a**) Changes in elastic modulus and Vickers hardness of α-Mg and alloys with different phases. (**b**) Changes in Poisson’s ratio and b/G ratio of α-Mg and alloys with different phases [42].

During the design of magnesium alloy components, first-principles calculations have been extensively used to investigate the physical properties of Mg–RE alloys, providing critical information that is often difficult to obtain experimentally. These insights help reveal the mechanical potential of different structures and establish a foundation for analyzing increasingly complex alloy systems. For example [42], a model of the β′-Mg_7_RE phase was constructed using first-principles methods to investigate the influence of RE elements on the elastic properties, electronic structure, and elastic anisotropy of alloys. The results, as shown in Figure 4, offer valuable references for tailoring alloy compositions to achieve desired performance.

To overcome the limitations associated with single alloying elements, the design of multicomponent alloy systems has emerged as an important strategy for performance enhancement. In quaternary Mg–Al–Zn–RE alloy systems [43,44], carefully selected combinations and proportions of elements can suppress the formation of brittle phases while leveraging nanoscale compound clusters to synergistically combine solid-solution and precipitation strengthening effects. In this system, the strong chemical affinity between Al and RE elements often results in the preferential formation of Al–RE intermetallic compounds. These compounds significantly reduce the solid solubility of RE elements in the magnesium matrix, thereby altering the dominant strengthening mechanisms. Thus, precise control over the Al-to-RE ratio is essential during alloy design to achieve a balance between precipitation and solid-solution strengthening.

First-principles calculations and thermodynamic phase diagram modeling provide powerful theoretical support for predicting phase stability in multicomponent alloys. Taking the Mg–Gd system as an example [45,46], when the Gd content exceeds 3 at.%, the Mg_5_Gd phase becomes the dominant strengthening precipitate. The stability of this phase is attributed to electronic hybridization between Gd 4f orbitals and Mg 3s orbitals, which enhances bonding energy and thermal stability. Furthermore, CALPHAD-based phase diagram calculations have shown [39,44,46] that the Mg_41_RE_5_ phase has the lowest Gibbs free energy in the Mg–RE system, indicating that it is thermodynamically the most stable; in contrast, Mg_12_RE phases (e.g., Mg_12_Y) are typically metastable and form only under specific aging conditions. Therefore, by tailoring heat treatment protocols—such as artificial aging—one can precisely control the type, size, and distribution of precipitates at the microscale, further enhancing overall material performance.

To establish more predictive models for alloy performance, researchers have developed multicomponent strength–ductility prediction frameworks that integrate various mechanisms including precipitation strengthening, solid-solution strengthening, and grain boundary strengthening. The Hall–Petch equation (σ_y_ = σ_0_ + K_y_∙d^−1/2^) is widely used to evaluate the contribution of grain refinement and dislocation strengthening to yield strength, where σ_y_ is the yield strength, σ_0_ is the lattice friction resistance for dislocation motion, K_y_ is the material constant, and d is the average grain diameter. According to this model [44], nanoscale precipitates such as MgZn_2_ can significantly improve strength via the Orowan bypass mechanism, contributing approximately 80 MPa to the total strength. On the other hand, solid-solution strengthening is often estimated by the following model: $∆\tau_{ss}=\sum \beta G\varepsilon^{3/2}c^{1/2}$, where ε is the lattice misfit and c is the molar fraction of the solute element. Based on this model [45,46], Gd, which induces significantly more lattice distortion than Ce, provides a much stronger solid-solution strengthening effect. This disparity underscores the critical role of RE element selection and proportioning in magnesium alloy design.

Additionally, the ability of RE elements to regulate grain and phase boundaries constitutes another key mechanism for enhancing the performance of multicomponent magnesium alloys. In these alloys, RE elements tend to segregate at grain boundaries, reducing grain boundary energy and suppressing the nucleation and growth of brittle phases (e.g., β-Mg_17_Al_12_). As found in Mg–9Al alloys, the addition of La leads to La–Al clustering at grain boundaries, which effectively hinders the precipitation of β-Mg_17_Al_12_. It has been reported [1,2,4] that this regulation significantly reduces the stress corrosion cracking rate in corrosive environments—by up to 60%.

Atomistic simulations have further revealed the mechanisms by which second phases interact with dislocations to enhance strength. Molecular dynamics simulations [43,44] show that when MgZn_2_ precipitates reach a radius of ~10 nm, they effectively obstruct dislocation motion via the Orowan mechanism, with dislocations forming typical loop structures around the particles. The critical shear stress increment Δ_τ_ is related to the inter-precipitate spacing λ by ${∆}_{\tau }=\frac{Gb}{2\pi \lambda }ln(\frac{2r}{b})$. When λ falls below 50 nm, this mechanism can contribute up to 150 MPa in shear stress, making it a major source of alloy strengthening.

However, under high-temperature conditions, the interfacial stability of the alloy tends to deteriorate due to accelerated grain boundary migration and precipitate coarsening. Related studies have indicated [45,46] that above 400 °C, RE segregation at grain boundaries weakens, leading to desorption of RE atoms from interfaces. This results in rapid grain growth through recrystallization and significantly reduces the structural stability and mechanical properties of the alloy.

The improvement of heat resistance in magnesium alloys is directly related to the effective content of alloying components. Increasing the content of specific components leads to higher precipitation densities of heat-resistant phases, which in turn improves heat resistance of magnesium alloys. However, increased strengthening phase density can also cause internal segregation and grain boundary embrittlement, reducing the ability of the material to accommodate intergranular deformation during plastic strain, increasing stress concentration, and greatly enhancing the probability of microcrack initiation and propagation. Macroscopically, this manifests as decreased ductility. From the perspective of composition, the trade-off between ductility and heat resistance is difficult to reconcile. The alloying content cannot be continuously increased, as the deterioration of ductility and increased cost diminish practical value, which is also one of the important factors limiting the development of heat-resistant magnesium alloys. Current strategies for improvement include (1) multi-element alloying for composition optimization, which utilizes interactions among alloying elements to improve precipitation efficiency, thereby reducing overall usage and indirectly enhancing ductility (e.g., WE series alloys, Mg–Al–RE–Ca alloys). However, this method has an upper limit and cannot fundamentally change alloy properties; (2) microstructural refinement through heat treatment or plastic deformation, which improves the morphology, size, and distribution of precipitates. Such treatments usually enhance mechanical properties, but their potential for property improvement is limited by the optimal processing parameters; (3) exploration of new strengthening mechanisms, often involving novel heat-resistant phases introduced through new elements or alloying formulations. This approach is the main driver of future progress but is usually associated with high cost and low efficiency using conventional research methods.

## 3. Application and Performance of RE Elements in Mg Alloys

### 3.1. Overview of RE Applications in Mg Alloys

**Figure 5:**
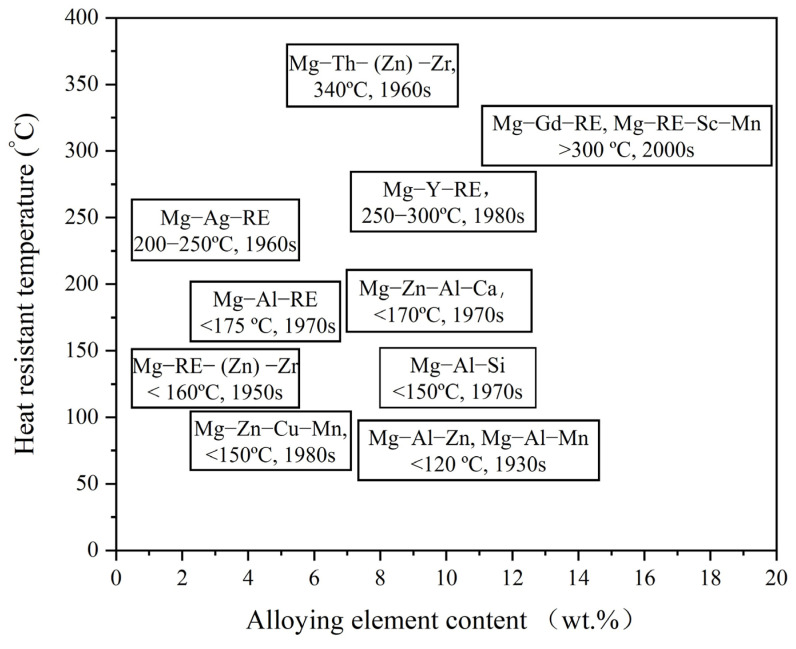
The development eras, compositional systems, and heat-resistant temperatures of early heat-resistant magnesium alloys [47].

The development timeline, compositional systems, and heat-resistance temperatures of early heat-resistant magnesium alloys are illustrated in Figure 5 [47]. Regarding elevated-temperature mechanical performance, researchers explored various alloy systems, and the compositions and properties of these alloys were closely tied to the research achievements of their respective eras. Most current heat-resistant magnesium alloys have evolved from these foundational systems. It is worth noting that the engineering application of heat-resistant magnesium alloys should consider not only mechanical properties but also material cost and processing complexity.

There are two main ways in which RE elements are applied in magnesium alloys: used as the modifiers to add into well-established alloy systems (e.g., improving the mechanical properties of Mg–Al and Mg–Zn alloys through RE addition), and as the principal alloying elements in systems such as Mg–Al–RE and Mg–RE. With different types and amounts of REs, these alloys exhibit distinct mechanical behaviors. The strengthening mechanisms and RE combinations vary significantly across alloy systems, highlighting the versatile roles of REs in magnesium alloy design.

In the early stages of magnesium alloy development, the specific mechanisms by which RE elements affected alloy properties were not fully understood. Although RE elements were introduced due to their unique alloying potential, many challenges hindered their practical application. For instance, ZM6 (Mg–Nd–Zr) type alloys with mixed REs suffered from coarse grains and inhomogeneous microstructures, which made them highly susceptible to hot cracking and stress concentration [47]. Moreover, the diverse chemical reactivities of individual elements within mixed RE elements led to complex behaviors in the melt, causing instability in alloying effects and limiting their engineering applications.

A breakthrough occurred in the 1940s when it was discovered that trace amounts of Zr added to Mg–RE alloys could act as heterogeneous nucleation sites, significantly increasing nucleation rates. This innovation suppressed coarse grain formation and enhanced high-temperature stability and overall mechanical properties. Based on this discovery, the EK-type (Mg–RE–Zr) alloy system was developed. These alloys exhibited exceptional strength, toughness, and processability, leading to their successful adoption in aerospace engine components—a milestone marking the industrialization of RE-containing magnesium alloys. Similar studies found that introducing RE elements into Mg–Zn alloys could greatly improve casting performance, creep resistance, and thermal stability. This helped address issues in traditional Mg–Zn alloys, such as hot-cracking susceptibility and flammability caused by low-melting-point phase precipitation. Based on this insight, ZE41 and EZ33 alloys were developed. These alloys not only offer excellent room-temperature strength and ductility but also exhibit good formability, making them widely used in aerospace, nuclear, and other high-tech fields.

By the 1970s, researchers had further expanded the scope of RE application in magnesium alloys. Studies revealed that adding light RE elements (e.g., Ce, La) to Mg–Al alloys can significantly improve creep resistance of the materials, particularly in high-temperature dynamic environments such as engine systems. Therefore, the AE-series alloys (e.g., AE42, AE41, AE21) were developed with a combination of good room- and high-temperature performance and became the first magnesium alloys applied in automotive transmission housings. Simultaneously, significant progress was made in applying heavy RE elements. Notably, adding Y to magnesium alloys through the Orowan mechanism can significantly improve yield strength and high-temperature performance. Based on this principle, high-strength and heat-resistant WE-series alloys were developed (e.g., WE43, WE54).

By the 1990s, research on RE magnesium alloys had entered a more systematic and mechanism-driven phase. Researchers began integrating computational thermodynamics, first-principles calculations, and advanced microscopy techniques to investigate the solid-solution and precipitation strengthening mechanisms of RE elements, while also exploring synergies with other strengthening elements such as Ag, Sc, and Zn. This led to the development of novel alloy systems, including Mg–Y–Gd, Mg–Gd, and Mg–Sc. These new alloys not only outperformed traditional Mg–RE alloys in room-temperature tensile strength but also exhibited superior high-temperature creep strength and thermal stability, gradually emerging as representatives of next-generation high-performance magnesium alloys.

In low-RE-content alloys (≤4 wt.%), strengthening mechanisms often rely on the formation of high-melting-point intermetallic compounds with Al. For example, AE-series alloys (e.g., AE42, AE44) utilize the formation of Al_11_RE_3_ phases with Ce, La, and Pr to effectively suppress the precipitation of low-melting-point Mg_17_Al_12_ phases, thereby improving thermal stability and corrosion resistance. In the Mg–RE–Zr system (e.g., EK30, EK31), mixed REs rich in Nd are typically used at total concentrations below 3 wt.%. This system excludes other primary alloying elements and instead relies on the precipitation of high-melting Mg–RE phases and Zr-induced grain refinement for strengthening.

The Mg–Zn–RE system (e.g., ZE33, ZE41, ZE63) primarily enhances strength through recrystallized microstructures induced by plastic deformation and the formation of LPSO phases. LPSO phases not only improve room-temperature strength but also enhance high-temperature mechanical performance by pinning microstructural features. Additional Zr further refines grain structure and improves the comprehensive mechanical properties of the alloys.

In the Mg–Ag–RE system (e.g., QE21, QE22, EQ21), Ag is introduced into Mg–RE–Zr alloys to form thermally stable dispersed secondary phases. These phases significantly improve mechanical performance under high-temperature conditions. Moreover, Ag enhances age-hardening behavior by accelerating the precipitation of strengthening phases and optimizing microstructural morphology.

High-RE-content alloys, such as those based on Mg–Y and Mg–Gd–Y (e.g., WE54, WE43, GW63, GW83), achieve pronounced age-hardening effects through the supersaturated solid solution of RE elements followed by the formation of high-melting-point RE compounds during aging. These alloys exhibit outstanding high-temperature strength, creep resistance, and thermal stability, representing the most advanced and high-performance RE-containing magnesium alloys available today.

### 3.2. Properties and Development of Mg-Al-RE Alloy

In the high-temperature strengthening mechanisms of Mg–Al-based alloys, the introduction of REs plays a crucial role in significantly enhancing both thermal stability and mechanical properties. Within this alloy system, RE elements mainly serve two key functions: first, they preferentially react with Al to form thermally stable, dispersed strengthening phases such as Al_11_RE_3_; second, they act as effective grain refiners, promoting a more uniform and finer microstructure, thereby improving the overall structural stability of the alloy. Compared with the traditional cubic Mg_17_Al_12_ phase, the Al_11_RE_3_ phase has a tetragonal structure with high atomic packing density and strong covalent bonding, resulting in a higher melting point and excellent thermal stability. As shown in Table 1, the melting point of the Al_11_RE_3_ phase typically exceeds 1000 °C, nearly three times higher than that of the Mg_17_Al_12_ phase, making it an ideal strengthening phase for high-temperature service conditions.

Based on this, Mg–Al–RE alloy systems have demonstrated superior high-temperature strength and creep resistance, maintaining good performance at 150–200 °C and outperforming aluminum alloys at elevated temperatures [48,49,50,51,52,53]. For instance, in traditional AZ-series alloys, the Mg_17_Al_12_ phase softens above 120 °C. However, the addition of 1.5–3.0 wt.% light REs (Ce/La/Nd) can effectively pin grain boundaries at high temperatures, thereby improving the mechanical properties under such conditions [48,49,50,51,52,53]. In a Mg–9Al–1.5Ce–1.0Nd alloy [53], when the volume fraction of Al_11_RE_3_ increases to 8.3%, the yield strength at 200 °C rises from 85 MPa to 145 MPa—a 70% improvement. This performance enhancement not only results from the stability of the phase itself at high temperatures, but also from the low diffusion coefficient of RE elements in the magnesium matrix, which significantly suppresses the coarsening and growth of precipitated phases, thereby extending service life.

To further improve the stability of the Al_11_RE_3_ phase, researchers have employed multi-RE composite strategies to enhance mechanical properties [51,52,53,54]. By co-introducing Ce and Nd to form an Al_11_(Ce, Nd)_3_ composite phase [52], a core–shell structure is achieved in which Ce is enriched in the core and Nd is distributed in the shell. This compositional gradient significantly lowers interfacial energy and effectively impedes rapid atomic migration at the phase boundary, thus enhancing thermal stability [52,54,55]. Transmission electron microscopy (TEM) observations show no significant coarsening of the composite RE phase even after exposure to 300 °C, offering a new pathway for improving the high-temperature performance of Mg–Al–RE alloys [56,57].

However, the strengthening effect of RE elements has a critical limit [53,58,59]. When the total RE content exceeds 3.5 wt.%, the Al–RE phases coarsen from their original fine size (~0.8 μm) to over 2.5 μm, substantially increasing microstructural brittleness and significantly reducing impact toughness. Therefore, in the optimization of Mg–Al–RE alloy composition, the RE content is typically controlled within 1.5–3.0 wt.%, and the total should not exceed 6 wt.% to balance strengthening and toughness.

**Figure 6:**
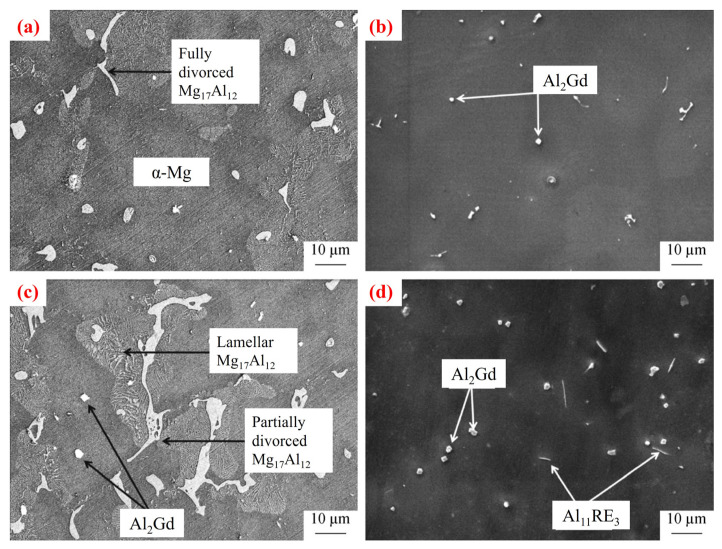
SEM images of microstructures of different as-cast alloys: (**a**) Mg-9Al, (**b**) Mg-4Al-2Gd, (**c**) Mg-9Al-2Gd, (**d**) Mg-4Al-1MM-1Gd [60].

For heavy RE elements, such as Y, Gd, and Er, their application is limited by metallurgical compatibility [37,38,49]. For example, the Al_2_Y phase formed from Y and Al often exceeds 5 μm in size and tends to segregate, reducing alloy density and increasing casting defects. Thus, the Y content should be strictly limited to 0.15–0.25 wt.%. When Gd is added to Mg–Al alloys [60], a rectangular precipitate phase identified as Al_2_Gd forms, as shown in Figure 6. Due to the higher crystallization temperature of Al_2_Gd compared to Al_11_RE_3_, more Al is consumed in forming Al_2_Gd, thereby reducing the availability of Al to form Al_11_RE_3_. The addition of mixed REs and Gd also depletes β-Mg_17_Al_12_ phase formation. For example, in Mg–9Al alloys, the volume fraction of Mg_17_Al_12_ decreases from 15.36% to 9.92% upon the addition of 2 wt.% Gd, while Al_2_Gd accounts for 2.96 vol.%. Notably, even small amounts of Y and Gd can effectively refine grains and suppress Mg_17_Al_12_ precipitation, demonstrating exceptional modification effects in AZ91 alloys [61,62,63,64]. However, when Y or Gd content exceeds a critical threshold (e.g., Y > 0.2 wt.%), the precipitation of coarse strengthening phases may instead degrade mechanical properties.

On this basis, to overcome the traditional belief that Al is detrimental to high-temperature performance in Mg alloys, researchers developed a Mg–3.5RE–1.5Gd–Mn–Al alloy [65]. Under creep conditions of 300 °C/50 MPa, its steady-state creep rate is as low as 1.35 × 10^−10^ s^−1^—71% lower than that of a counterpart alloy without Al, indicating outstanding creep resistance. This improvement is mainly attributed to the formation of AlMnGd short-range ordered (SRO) structures and 2–10 nm nanoclusters, which interact with dislocations in the matrix, significantly pinning them and impeding plastic deformation during creep. Moreover, a network structure of Mg_12_RE phases effectively suppresses grain boundary sliding and diffusion, enhancing overall structural stability. Al does not precipitate as Mg_17_Al_12_ during high-temperature creep, but instead preferentially forms thermally stable Al_2_RE_3_ compounds with Gd, exhibiting excellent anti-coarsening ability and supporting stable service at 300 °C. This finding challenges the traditional view that Al impairs high-temperature properties in Mg alloys and provides a new strategy for designing high-temperature Mg–Al-based alloys.

In another Mg–5Gd–1.5Sm–0.7Al alloy (HPDC) modified with low Al [66], the yield strength reached about 200 MPa, and the elongation about 8.5%, demonstrating excellent strength–ductility synergy. Research has shown that the alloy contains two types of α-Mg grains: coarse-grained α_1_-Mg and finely dispersed α_2_-Mg. High-angle annular dark field scanning transmission electron microscopy (HAADF-STEM) analysis showed that after aging treatment, dense β′ precipitates and chain-like β′ structures were formed near grain boundaries, while ultrafine β″-(Mg,Al)_3_Sm precipitates and Al-(Gd,Sm) clusters formed at the grain centers. Relatively small grains, Al-(Gd,Sm) primary particles, solute segregation near grain boundaries, and various types of precipitates all contribute to improving alloy strength. In addition, the multi-scale α-Mg grains, various intermetallic compounds, and discontinuous skeleton structures of different precipitates in the microstructure also endow the material with excellent ductility.

The room-temperature mechanical properties of Mg–Al–RE alloys are shown in Table 2. Each alloy is sorted by Al content, rare earth type, and content. It can be observed that, based on the processing characteristics and practical production requirements of Mg–Al alloys, most are produced by the high-pressure die casting. The mechanical performance is closely related to RE content. The combined application of different RE elements, or the addition of REs together with other alloying elements, yields better properties at the same content levels [67,68,69,70,71]. The strengthening effect of a single RE element typically exhibits an optimal concentration, generally around 4 wt.% for most light REs. Beyond this threshold, coarse precipitates form, increasing microstructural brittleness and degrading alloy performance. Additionally, as RE content increases, phase coarsening occurs, while density remains largely unchanged, which does not enhance high-temperature performance despite further RE additions. Therefore, the RE content in Mg–Al alloys is generally kept below 6 wt.% to avoid these adverse effects.

Currently, Mg–Al–RE alloys have achieved thermal stability and service limits of up to 250–300 °C, within which their mechanical performance typically surpasses that of equivalent aluminum alloys. However, at temperatures exceeding 300 °C, the vacancy formation energy of interfacial strengthening phases such as Al_11_RE_3_ significantly drops, accompanied by a rapid increase in atomic mobility, leading to phase coarsening and weakened strengthening effects. Thus, further enhancing interface stability, optimizing RE distribution, and introducing novel clusters and short-range ordered structures are key strategies for achieving higher service temperatures.

### 3.3. Strengthening of Mg-Zn Alloy by RE Elements

Mg-Zn alloys have attracted significant attention in biomedical implants and precision casting due to their excellent biocompatibility and moderate mechanical strength. However, their limited high-temperature strength—characterized by a sharp decline above 150 °C—and poor creep resistance have long constrained their broader application. The addition of RE elements addresses these limitations by forming thermally stable multicomponent intermetallic compounds with Zn. These dispersed phases not only retain their strengthening effect at elevated temperatures but also enhance hardness and thermal strength through lattice distortion and interfacial strengthening. Incorporation of RE elements typically increases the service temperature of Mg-Zn alloys by 30–50 °C [78,79,80,81,82,83].

Furthermore, the addition of RE elements reduces the strong basal texture commonly observed in Mg alloys, promoting activation of non-basal slip systems and thereby significantly improving plastic formability. This is particularly beneficial for applications requiring high ductility and formability, such as sheet metal stamping [84,85,86]. Nevertheless, the quantity and size of strengthening precipitates must be precisely controlled. Excessive amounts can hinder grain boundary sliding, compromise ductility, and limit overall performance.

In the Mg-Zn-Y alloy system [78,79,80,81,82,83], the addition of 2 wt.% Y facilitates the formation of two key strengthening phases. The first is the intragranular I-phase (Mg_3_Zn_3_Y_2_), a quasicrystalline phase with particle sizes ranging from 10 to 30 nm and a high melting point of ~620 °C—well above that of conventional Mg-Zn phases (~425 °C). The I-phase exhibits a semi-coherent interface with the matrix, conferring excellent thermal stability; even after 50 h of exposure at 300 °C, its particle size remains within 35–50 nm, with a volume fraction exceeding 12%. The second is the W-phase (Mg_3_Zn_2_Y_3_), distributed discontinuously along grain boundaries with particle sizes between 0.2 and 0.8 μm. This phase effectively pins grain boundaries, suppressing grain growth and recrystallization, thereby delaying softening at elevated temperatures.

**Figure 7:**
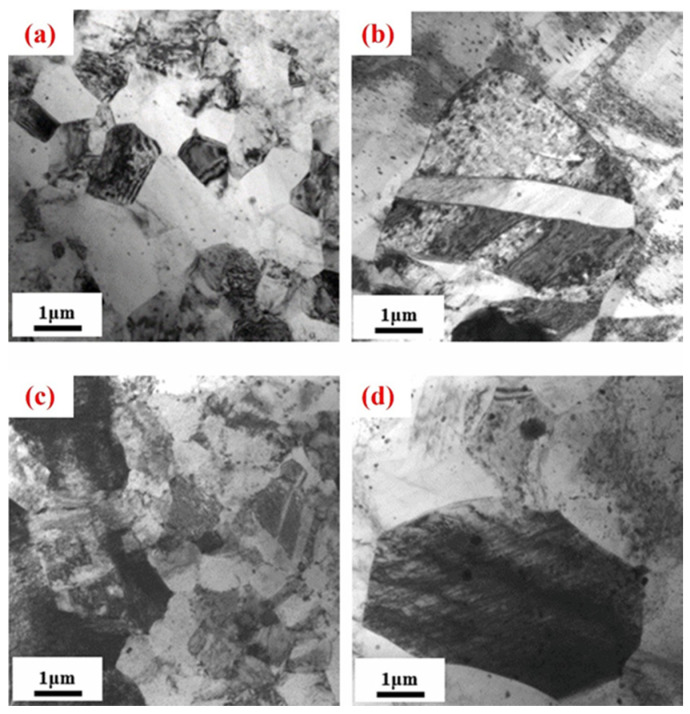
TEM images of DRX grains in Mg-5.9Zn-1RE-0.6Zr alloy after different peak aging treatments: (**a**) 200 °C × 72 h, (**b**) 300 °C × 24 h, (**c**) 400 °C × 24 h, (**d**) 450 °C × 24 h [87].

Figure 7 illustrates the DRX grain morphology of a Mg-5.9Zn-1RE-0.6Zr alloy under various peak aging conditions [87]. After aging at 200 °C, DRX grain size remained stable. With increasing aging temperature, grain size increased from the nanometer to micrometer scale. Notably, above 300 °C, extensive twinning was observed, accompanied by rapid grain growth via boundary migration. Experimental results indicated that alloys aged above 300 °C exhibit enhanced elongation, suggesting that twinning mechanisms, activated by RE additions, contribute positively to ductility and structural stability.

The high-temperature performance of Mg-Zn-RE alloys is highly dependent on the type and proportion of RE elements. For alloys dominated by Zn or containing >2 wt.% Zn, room-temperature mechanical properties are summarized in Table 3. The alloys are arranged in the order of Zn content and rare earth content. As shown, plastic forming is the preferred process for most Zn-containing Mg alloys, and strength and ductility are not directly related to the total alloying content but to the types of REs, specific combinations of REs, and Zn/RE ratio. Light REs such as Ce and La are more suitable for service conditions below 250 °C, where they rapidly form nanoscale strengthening precipitates that enhance strength in the extruded state. In contrast, heavy REs such as Y and Gd are more appropriate for applications above 300 °C due to their solute drag effects and ability to enhance microstructural stability.

Studies [98,99] have shown that an optimal balance between strength and ductility is achieved when the light-to-heavy RE ratio is maintained between 1:1 and 2:1. Zn content also plays a critical role; maintaining it within 3–5 wt.% promotes the formation of fine and uniform eutectic structures (e.g., MgZn + MgZnRE), enhancing resistance to thermal softening and creep. However, exceeding 6 wt.% Zn leads to precipitation of coarse and brittle Mg_7_Zn_3_ phases, which reduce microstructural stability and introduce stress concentrations that accelerate failure.

In addition, the thermodynamic and mechanical properties of Mg-Zn-RE intermetallic compounds have been revealed through first-principles calculations [100,101]. Among them, Mg_2_La exhibits the lowest formation energy, indicating superior thermodynamic stability; MgZn_2_ demonstrates excellent ductility and anisotropy matching; Mg_2_Y and MgZn_2_, based on elastic modulus analyses, behave as ductile materials and are thus suitable for enhancing toughness. Thermodynamic data further indicate that the Gibbs free energy of these phases decreases with increasing temperature, while heat capacity increases, suggesting their potential for long-term service at elevated temperatures.

Beyond alloy composition optimization, thermomechanical processing plays a decisive role in achieving desirable performance in Mg-Zn-RE alloys. Due to the tendency of Mg alloys to undergo dynamic recrystallization and texture development at elevated temperatures, their traditional extrusion rates are much lower than those of Al alloys, hindering industrial scalability. Nevertheless, through compositional fine-tuning (e.g., optimizing Zn and RE contents) and process control (e.g., deformation temperature and extrusion ratio), recent studies have increased extrusion speeds of certain Mg-Zn-RE alloys from the conventional 10–15 m/min to 30–60 m/min—while maintaining uniform microstructures and fine precipitate distributions, thus meeting industrial processing demands.

Post-processing heat treatments (e.g., T5, T6) can further enhance mechanical properties. For example, forged and aged ZK60-RE alloys have achieved yield strengths exceeding 500 MPa with elongation above 8%, reflecting a successful synergy between strength and ductility. In as-cast conditions, Mg-Zn-RE alloys typically exhibit a ternary eutectic network microstructure, such as the Mg_12_(RE, Zn) phase. These uniformly distributed eutectic networks refine the grain size to ~30 μm and contribute to high-temperature stability and fracture toughness. The fracture behavior is predominantly intergranular, indicating higher energy absorption capacity compared to traditional alloys—demonstrating superior structural reliability and service stability. This eutectic structure remains stable between 200 and 300 °C and is minimally affected by heat treatment, offering advantages in post-processing cost and efficiency.

Despite these advancements, the engineering application of Mg-Zn-RE alloys still faces several technical challenges. On one hand, strengthening phases tend to coarsen at elevated temperatures, diminishing their efficacy. On the other hand, excessive precipitation of secondary phases may cause stress concentration and degrade ductility, while inhomogeneous composition and difficulties in microstructural control limit performance consistency in large-scale production. To address these issues, researchers are exploring multiple strategies, including improving RE utilization efficiency, developing controlled precipitation and distribution mechanisms, establishing multiscale synergistic strengthening models, and formulating efficient heat treatment protocols. Moreover, the design of multicomponent alloys that combine the Mg-Zn-RE system with other strengthening mechanisms (e.g., Mg-Sn, Mg-Ca, Mg-Bi), or the incorporation of ceramic nanoparticles (e.g., SiC, AlN) to enhance phase stability and interfacial bonding, represent promising research directions. Ultimately, the goal is to develop a high-performance Mg-Zn-RE alloy system with high strength, excellent ductility, superior thermal stability, and good formability, capable of withstanding complex service environments over extended periods.

### 3.4. Development and Limitations of Mg–RE Alloys

Mg–RE alloys are considered one of the most promising heat-resistant systems among magnesium-based materials, with their performance evolution demonstrating the pivotal role of RE elements in both microstructural refinement and macroscopic mechanical enhancement. Early generations of heat-resistant Mg alloys, represented by binary Mg-Y systems, typically exhibited thermal stability limited to approximately 200 °C. However, with the advancement of alloy design principles and a deeper understanding of the multifaceted strengthening mechanisms associated with RE elements, modern high-performance multicomponent systems such as Mg-Gd-Y-Nd-Zr have extended the upper service temperature to around 350 °C. This progress represents a systematic breakthrough not only through compositional complexity but also through synergistic optimization of precipitation behavior, texture control, and forming mechanisms.

The fundamental strengthening mechanism of RE elements lies in their ability to form thermodynamically stable, ultra-fine, and uniformly dispersed secondary phases with Mg, including Mg_12_RE, Mg_3_RE, β′ (Mg_7_RE), and γ″ (Mg_5_RE). These particles obstruct dislocation motion via the Orowan mechanism, increasing resistance to plastic deformation, and act as pinning sites at grain boundaries, thereby inhibiting grain boundary sliding and coarsening. Such effects confer exceptional strength and thermal stability to the alloy.

**Figure 8:**
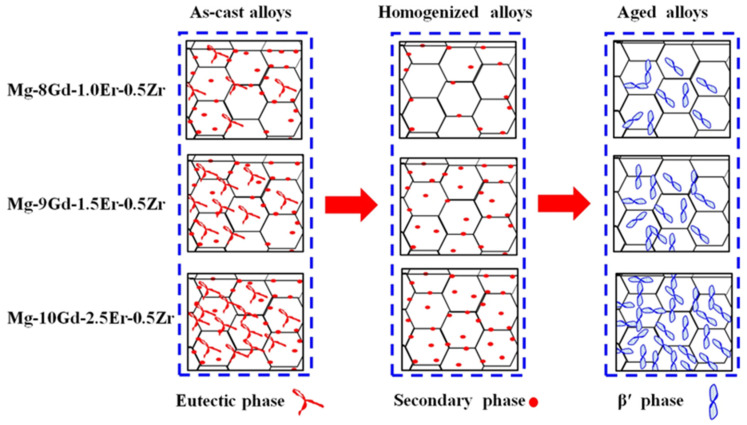
Schematic diagram of precipitation strengthening evolution process of Mg-Gd-Er-Zr alloy [102].

For example [102], in the Mg-Gd-Er-Zr system, the volume fraction of eutectic phases in the microstructure is positively correlated with RE content, enhancing hardness and strength. After T4 solid solution treatment and subsequent aging, the eutectic RE phase dissolves into the matrix, promoting the reformation of β′ precipitates in a high-density and uniformly distributed form. As shown in Figure 8, the RE precipitate phases significantly improved tensile properties. The alloy yield strength (YS) increased from 158 MPa (pre-aged condition) to 245 MPa, while the ultimate tensile strength (UTS) rose from 254 MPa to 330 MPa. The elongation at break (EL) decreased from 12% to 2.0%.

As summarized in Table 4, each alloy is listed by element type and content. In alloys with RE elements as the main alloying elements, mechanical properties are directly related to RE content, with strength increasing and ductility decreasing. High-RE-content Mg alloys, particularly those alloyed with Gd and Y, demonstrate a favorable balance between strength and toughness. In the Mg-Gd-Y-Zr system [1,3,5,97,103,104], precipitation behavior during multi-stage aging exhibits a three-stage evolution: (1) formation of ~1–2 nm Guinier–Preston (GP) zones at 150 °C, providing initial strengthening via coherent strain fields; (2) transformation into β′ phases at 200 °C, preferentially oriented along the <11–20> direction of the matrix and forming helical dislocation loops during bypass; and (3) subsequent growth into γ″ phases at 250 °C, yielding a stable core–shell precipitate structure. This multi-scale precipitate architecture enables Mg-12Gd-3Y-0.5Zr to achieve a room-temperature yield strength of 380 MPa and retain 280 MPa at 300 °C [104], with creep resistance superior to most cast aluminum alloys, underscoring its potential for high-temperature applications.

In terms of plastic deformation, RE elements inhibit dynamic recrystallization through multiple mechanisms, enhancing hot workability. Specifically, RE solutes reduce the stacking fault energy of the Mg matrix, promoting partial dislocation formation and increasing cross-slip frequency, thereby delaying recrystallization [5]. Additionally, RE elements weaken the strong basal texture of Mg alloys and activate non-basal slip systems, improving homogeneous deformation, reducing anisotropy, and raising forming limits. Through solid-solution strengthening and grain refinement, RE additions also enhance low-temperature workability, providing an effective strategy to balance strength and ductility [126,127,128,129].

As shown in Table 5, alloys with lower RE content benefit from hybridization strategies involving light and heavy RE elements, maintaining the total RE content within 6–10 wt.% to achieve fine-scale multi-element alloying. This approach effectively suppresses coarse RE-rich phases and prevents embrittlement, thereby improving the strength–ductility synergy. From the perspective of strengthening mechanisms, the performance of Mg–RE alloys arises from the combined effects of solid-solution strengthening, precipitation strengthening, grain boundary strengthening, and texture optimization. The relative importance of these mechanisms in specific applications must be evaluated based on alloy composition and processing history. Recent studies [103,130,131,132,133,134,135] have shown that, in Mg-Gd-Y-Zn-Zr alloys, the initial deformation microstructure has limited effect on precipitation during aging but plays a crucial role in controlling subsequent recrystallization and ductility. High dislocation densities introduced via deformation promote static recrystallization and basal slip activation, thereby enhancing ductility without sacrificing strength. In the context of strengthening modeling, studies have indicated [136,137,138,139] that conventional Orowan-based models may underestimate the strengthening contribution of β′ phases. Updated models incorporating precipitate shearing mechanisms provide a more accurate assessment. Furthermore, the development of high-aspect-ratio, oriented precipitate structures—such as β_1_ prismatic plates and 14H LPSO basal lamellae—has shown promise in forming ultra-dense strengthening architectures, achieving superior strength, creep resistance, and thermal stability.

Beyond traditional alloying strategies, emerging approaches such as bimodal grain structures, pre-twinning-induced strengthening, and nano-twin architectures are advancing the realization of strength–ductility synergy [141,142,143]. By tuning the critical resolved shear stress difference between basal and non-basal slip systems, alloy plasticity can be significantly improved. Coupled with advanced severe plastic deformation techniques such as equal channel angular pressing (ECAP) and high-pressure rolling (HPR), these methods facilitate texture homogenization and twinning network formation, effectively overcoming the conventional trade-off between strength and ductility in Mg alloys.

### 3.5. Multicomponent Magnesium Alloys Containing REs

In the study of heat-resistant magnesium alloys, the diversification of RE elements has been widely demonstrated as an effective strategy to enhance high-temperature mechanical performance. Multi-element microalloying is generally a simple and effective approach. However, this method is most effective for alloys containing a single RE element at high content, such as replacing Nd in WE43 with a Nd-Gd mixture or substituting Y with yttrium-rich RE. These modifications typically improve alloy plasticity and the strengthening effect of heat treatment. Nevertheless, this improvement has an upper limit and cannot fundamentally alter alloy properties or significantly change the precipitation sequence.

Beyond the intrinsic synergistic strengthening among RE elements, increasing attention has been directed toward the combined addition of REs with other alloying elements. Such multicomponent alloying aims to achieve more efficient strengthening than single-element systems by modulating precipitation behavior, interface stability, and microstructural evolution through the cooperative effects of elements such as Ca, Sc, Sr, Si, Mn, and Ag [1,2,3,4,5,6,39,43,48]. As shown in Table 6, each alloy is listed according to the types and amounts of diversified elements. The development of novel heat-resistant Mg–RE alloys based on multicomponent alloying has extended the application boundaries of these materials in high-temperature environments, marking a shift from single-element optimization toward multi-element synergy.

**Figure 9:**
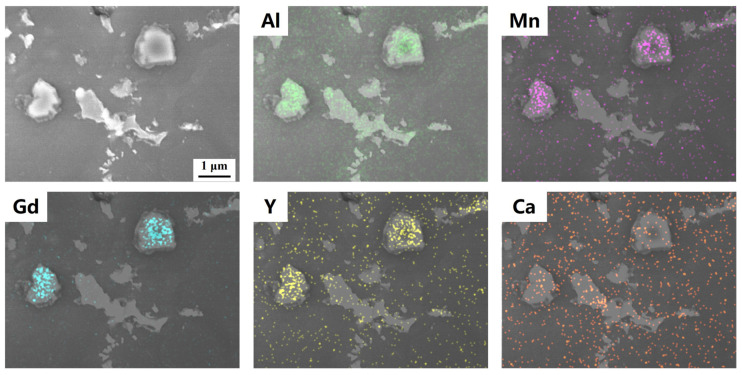
Element distribution of the AM60 + 0.2RE + 0.2Ca alloy [6].

In the Mg-RE-Ca alloy, Ca plays a pivotal role in precipitation strengthening and interface stabilization while significantly influencing the spatial distribution of RE elements. Ca preferentially segregates to grain boundaries, driving RE elements toward grain interiors and enhancing solid-solution strengthening. Ca and RE elements also co-participate in forming thermally stable intermetallic compounds such as (Mg,Ca)_5_RE at grain boundaries and within the matrix. These compounds maintain structural integrity at temperatures up to 400 °C, effectively pinning dislocations and grain boundaries and delaying softening [145]. In addition, the synergistic effect between Ca and REs significantly improves oxidation resistance, attributed to the rapid formation of a dense protective oxide film, which reduces oxygen diffusion and enhances self-healing efficiency, thus improving mechanical and flame-retardant properties [6]. In AM60 + 0.2RE alloys, a small addition of Ca resulted in a dispersed distribution throughout the microstructure, as shown in Figure 9, without aggregation or disruption of RE distribution. While Ca did not alter the morphology or size of RE-containing precipitates, RE elements did not inhibit the localized segregation of Ca to Mg-Al phases. These results indicate that composite alloying enhances flame resistance and microstructural refinement. Precise multicomponent optimization can improve precipitation efficiency, reduce total alloying content, and mitigate detrimental effects, ultimately enhancing overall alloy performance.

Kubásek et al. [148] reported that Mg-4.5Gd-3.4Y-2.6Ca alloy, with relatively high RE and Ca content, achieved an ignition temperature of 1100 °C. Formation of a dense (Y, Gd)_2_O_3_ surface oxide layer was identified as the primary factor in improving flame retardancy. As an extruded alloy processed at 350 °C, it exhibited a yield strength of ~230 MPa, a tensile strength of 300 MPa, and an elongation of 10%, showing superior ductility compared with other flame-retardant Mg alloys. Zhu et al. [149] demonstrated that alloying commercial WE43 with Ca significantly improved flame retardancy, primarily due to Ca-enhanced Y surface reactivity and the formation of a stable Y_2_O_3_ oxide layer.

Similarly to Ca, Sr in Mg-RE-Sr systems provides a unique strengthening pathway by modulating precipitation behavior. In the Mg-Nd-Sr system [60], Sr altered Nd precipitation from β-Mg_12_Nd to the more thermally stable Mg_41_Nd_5_Sr_2_ phase. This phase exhibits superior structural stability and interfacial bonding, effectively anchoring dislocations and grain boundaries at elevated temperatures and maintaining mechanical strength.

Si is also frequently employed in combination with REs to attain composite strengthening effects. While REs or Si individually may reduce ductility, their combination enhances precipitation efficiency, reducing total alloying content and indirectly improving ductility. In Mg-RE-Si systems [52,83], Y and Si form Y_5_Si_3_, a Laves phase with a high melting point (>1800 °C) and strong covalent bonding, providing excellent resistance to dislocation shearing. The fine, dispersed nature of Y_5_Si_3_ also inhibits grain growth and recrystallization. In situ TEM heating confirmed that Y_5_Si_3_ remains stable at 400 °C, significantly enhancing thermal stability and resistance to high-temperature deformation.

Among transition metals, Mn and Ag synergize with REs to enhance alloy properties. In Mg-Gd-Mn alloys [33,116,119], Mn reacts with residual Fe impurities to form α-Mn(Fe), mitigating Fe-induced degradation of thermal stability. Mn also forms short-range ordered clusters that, together with Gd solutes, create local diffusion barriers, suppressing nucleation of recrystallization and dynamic recrystallization, thereby improving thermal stability and ductility. Ag in Mg-RE-Ag systems [30,35,105,107,111,115,147,150,151] co-precipitates with REs to form nanoscale, high-hardness, thermally stable precipitates and segregates at grain boundaries to enhance stability. Ag-RE precipitates such as Mg_3_AgRE and Ag_4_RE form at low aging temperatures and retain long-term thermal stability, suitable for constructing multiscale strengthening architectures. Ag also suppresses grain boundary sliding, increases boundary strength, and reduces high-temperature plastic flow, improving creep resistance and fatigue performance. Mg-Gd-Y-Ag alloys achieve high yield strengths (>270 MPa) at 300 °C with lower creep rates than Ag-free counterparts. Composite alloying stabilizes secondary phases and affects diffusion kinetics and microstructural evolution. For example, in Mg-Gd-Y-Zr-Ag systems, Ag addition retards transformation from GP zones to β′ phases, extending the effective aging window and promoting stable strengthening across a wide temperature range. Zr, a conventional grain refiner, further improves recrystallization homogeneity and plastic formability when combined with Ag or Ca. These multicomponent synergistic mechanisms provide novel pathways for strength–ductility optimization and broaden the processing window for high-temperature forming. Certain multicomponent systems, such as Mg-RE-Ag-Si, show good high-temperature compatibility and allow multiphase precipitation without forming brittle phases, laying the foundation for ultra-high-strength Mg alloys.

## 4. Application and Development

In recent years, RE-containing Mg alloys have made continuous progress in engineering applications. These advancements primarily focus on improving component quality through optimized processing parameters and fabrication techniques, thereby fully exploiting the performance advantages of these alloys in high-end industrial sectors. In the aerospace industry, Mg–RE alloys have shown remarkable performance improvements in critical components through topological optimization and precision casting technologies [152,153,154,155,156,157]. For example, the main reduction gearbox housing of helicopters, manufactured from WE43 alloy via precision casting, exhibits a 40% higher specific strength compared with aluminum alloys. This component can operate continuously at 300 °C for over 500 h, while its weight was reduced from 12.3 kg to 8.5 kg [153,154,155]. Similarly, the ZM6 alloy (Mg-2.5Nd-0.5Zn-Zr) has been applied in civil aircraft for auxiliary power unit brackets. Through hot isostatic pressing, porosity was even reduced to below 0.3%, thereby extending fatigue life to 2.5 × 10^6^ cycles and achieving a 34% weight reduction relative to conventional materials [154]. Recent studies have shown that the WE43 alloy fabricated by selective laser melting (SLM) achieves a room-temperature tensile strength of 380 MPa, representing a 25% improvement over as-cast counterparts. This superior performance, surpassing that of aluminum alloys, has enabled its application in vector control mechanisms for rocket engines [154,155]. In the development of SpaceX’s next-generation interstellar spacecraft secondary engine, a combination of WE43B alloy integral casting and SLM technology was attempted for preparing the nozzle extension. The wall thickness of the component ranged from 3 mm to 0.8 mm, and the shape was optimized through topology. Compared with traditional nickel-based alloys, the component has achieved a weight reduction of up to 52%, demonstrating great potential for lightweighting [154,155]. In the communications sector, precision-forged Mg-Y-Nd alloys have been applied in base station cooling systems. These alloys were engineered into biomimetic honeycomb-structured cooling fins, delivering a 20% increase in thermal conductivity compared with aluminum alloys. Under a heat flux of 40 W/cm^2^, these systems-maintained chip temperatures below 85 °C, significantly enhancing device longevity [5,6,153,155].

Grain refinement plays a pivotal role in the mechanical performance of Mg–RE alloys. Although the industry-standard Zr inoculation technique is well established, its effectiveness is highly sensitive to melt purity, reaction byproducts, and external field conditions. To address these limitations, recent developments have integrated chemical inoculation with external physical fields such as ultrasonic vibration and electromagnetic stirring. These approaches have significantly improved the nucleation efficiency of Zr in RE-rich melts, enhancing grain refinement and mitigating issues such as insufficient heterogeneous nucleation and grain coarsening [158,159,160,161]. Additionally, to overcome challenges posed by complex casting geometries, such as thin walls and large temperature gradients, emerging manufacturing methods—including pressure-assisted solidification, investment casting, and 3D printing—have been adopted. These techniques not only improve formability and dimensional accuracy but also enable ultrafast cooling to promote metastable phase formation and microstructural refinement, offering novel routes for structural control.

**Figure 10:**
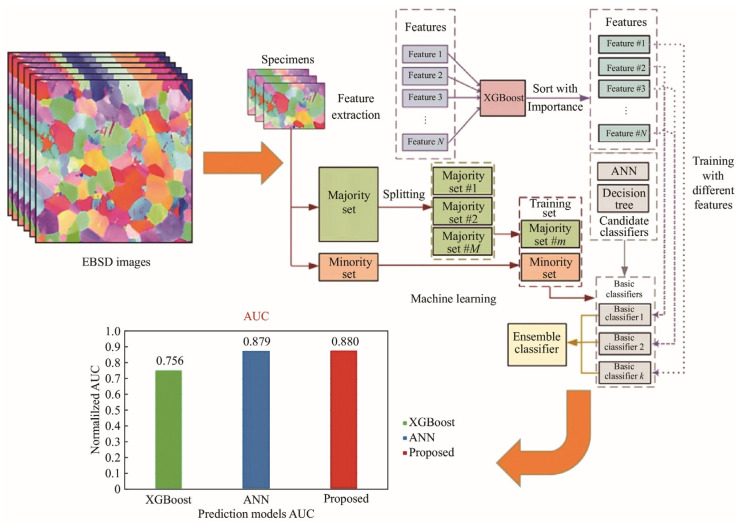
Prediction of twin nucleation in Mg–RE alloys using EBSD and machine learning methods [162].

**Figure 11:**
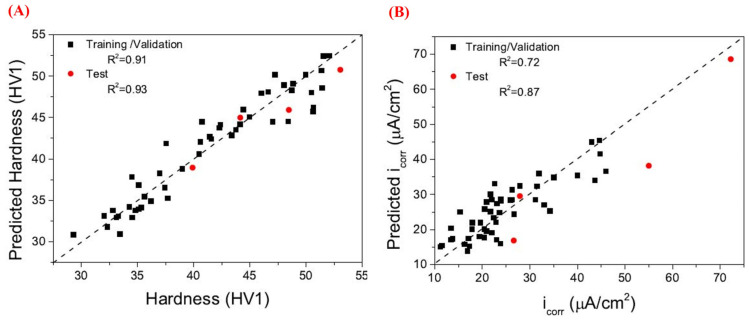
ANN-predicted versus observed hardness (**A**) and corrosion rate (**B**) for both the training/validation and test sets [164].

As the application of Mg–RE alloys expands, their complex microstructure–property relationships have rendered conventional experimental methods inadequate for comprehensive performance optimization and alloy development. In this context, the integration of machine learning and big data analytics has emerged as a powerful tool to enhance research efficiency [162,163,164,165,166]. Gui et al. [162] developed an analysis method combining machine learning and electron backscatter diffraction (EBSD) to predict twinning nucleation and other behaviors in Mg–RE alloys. As shown in Figure 10, a classification and regression tree (CART)-based ensemble algorithm was employed to forecast twin nucleation behavior in Mg-4Y-3Nd-2Sm-0.5Zr alloys. Twinning was detected in 68 out of 297 initial grains, and the influence of eight microstructural features—including grain size and Schmid factor—was systematically assessed. This study highlights the utility of combining machine learning with EBSD to understand deformation-induced twinning phenomena in Mg–RE alloys. The rapid advancement of materials genome engineering and machine learning has provided new avenues for the exploration of Mg–RE alloys. Data-driven approaches integrating theoretical modeling with analytical tools are proving increasingly effective in alloy design [163]. To manage large datasets and high-throughput simulations, machine learning models have been applied to map the relationships among alloy composition, microstructure, and performance. Techniques such as artificial neural networks (ANNs) and gene expression programming have enabled efficient predictions of mechanical properties and corrosion behavior in Mg–RE alloys. These models not only enhance prediction accuracy but also significantly reduce experimental costs and time. Xia et al. [164] conducted a systematic investigation into the effects of Zn, Ca, Zr, Gd, and Sr on the corrosion rate of magnesium alloys. Using five compositional features as inputs and incorporating hardness and corrosion rate as outputs, ANN models were developed for property prediction. As illustrated in Figure 11, the models accurately forecasted hardness and corrosion resistance, facilitating the design of high-strength, corrosion-resistant Mg alloys.

Despite these promising advances, several challenges remain in applying machine learning to Mg–RE alloy research. Key bottlenecks include limitations in data quality and model generalizability. Commercial databases often lack complete phase composition records, while microstructural datasets frequently contain missing parameters. To overcome these issues, several strategies have been proposed: (1) Development of high-precision automated characterization systems, such as EBSD–APT hybrid technologies, to improve microstructural digitization efficiency by an order of magnitude; (2) Construction of cross-scale correlation models integrating multilevel data from electronic structure (via DFT calculations) to mesoscopic microstructure (via phase-field simulations); (3) Application of transfer learning to adapt reinforcement models from aluminum alloys to magnesium systems, thereby enhancing prediction accuracy under limited data conditions.

These breakthroughs are expected to provide critical technological support for the development of next-generation heat-resistant magnesium alloys.

At present, the alloy design method based on artificial intelligence is gradually improving. Although the results are not yet comparable to those obtained from empirical approaches, effective primary screening can greatly enhance research efficiency, reduce the workload of testing and verification, and compensate for inaccuracies through manual correction and optimization. This should serve as an effective paradigm for future materials research. While extensive studies have been conducted on the high-temperature resistance mechanisms and performance of heat-resistant magnesium alloys, there remain significant gaps in the combined application of REs with other elements and in determining optimal multicomponent ratios. If traditional methods were used to fill these gaps, the costs would be substantial and the benefits uncertain. This underscores the value of machine learning as an auxiliary tool in current research on heat-resistant magnesium alloys. Filling these gaps can further elucidate the effects of individual components and optimize alloy composition. Through this approach, not only can higher-performance magnesium alloys be developed, but a data foundation and new technological pathways for exploring more accurate and effective strengthening mechanisms can also be well established.

## 5. Summary and Outlook

With the continuous advancement of aerospace, new energy, and other high-performance sectors, lightweight structural materials are increasingly required to combine superior heat resistance, flame retardancy, and comprehensive mechanical properties. The incorporation of RE elements has emerged as an effective strategy to meet these demands. Compared with other alloying elements, RE additions provide significant strengthening effects and superior overall performance, but the potential to improve material properties by increasing RE content is already very limited.

A key challenge for RE-containing magnesium alloys lies in balancing property enhancement with resource cost. The high price of heavy RE elements such as Y and Gd results in alloys like WE43 being several times more expensive than commercial AZ91 alloy. Addressing this issue requires improving strengthening efficiency through optimized combinations of RE elements and their synergistic interactions with other alloying elements. Advanced approaches such as machine learning offer systematic strategies for alloy design and optimization.

Moreover, with the rapid development of precision manufacturing techniques, particularly additive manufacturing, Mg–RE alloys must not only exhibit improved mechanical properties but also demonstrate enhanced processability. Optimization of both alloy composition and processing parameters is essential to adapt these alloys to future high-precision fabrication technologies. In addition, further improvements in corrosion resistance, flame retardancy, and biocompatibility are required to meet the expanding demands of aerospace, automotive, and biomedical applications.

Through the adoption of efficient research methodologies, continued exploration of strengthening mechanisms, and systematic compositional optimization, next-generation Mg–RE alloys can be developed with superior performance and manufacturability. Such progress will further promote their application in key industries and contribute to the broader goals of technological innovation and sustainable development.

## Figures and Tables

**Table 1:** Operating temperature and melting point of main strengthening phases of common RE magnesium alloys.

Mg Alloys		Operating Temperature	Strengthening Phases	Melting Point of Precipitation Phase
Mg-Al-RE	Mg-Al-La	<200 °C	Al_11_La_3_	1240 °C
	Mg-Al-Ce		Al_11_Ce_3_	1235 °C
			Al_2_Ce	1480 °C
	Mg-Al-Nd		Al_11_Nd_3_	1235 °C
			Al_2_Nd	1460 °C
	Mg-Al-Y		Al_2_Y	1485 °C
Mg-Zn-RE	Mg-Zn-Y	<175 °C	Mg_3_Zn_6_Y	475 °C
			Mg_12_ZnY	550 °C
			Mg_3_Zn_3_Y_2_	--
Mg-RE	Mg-La	<250 °C	Mg_17_La_2_	--
	Mg-Ce		Mg_12_Ce	610 °C
	Mg-Nd		Mg_12_Nd	560 °C
	Mg-Sm		Mg_41_Sm_5_	--
	Mg-Gd	<300 °C	Mg_5_Gd	640 °C
	Mg-Y		Mg_24_Y_5_	620 °C

**Table 2:** Tensile properties of typical Mg-Al-RE alloys at room temperature.

Alloys (wt.%)	Process	UTS (MPa)	YS (MPa)	EL (%)	Ref.
Mg-1.2Al-0.2Ce-0.4Ca	Extrusion	358	350	12.1	[48]
Mg-2Al-3Y-4Nd	Heat treatment	285	182	7.4	[49]
Mg-2.5Al-4La-0.5Mn	HPDC *	253	149	11.5	[50]
Mg-2.8Al-3.8La-0.4Nd	HPDC	240	137	8.8	[49]
Mg-4Al-3La-1Ca-0.3Mn	HPDC	274	220	6.3	[72]
Mg-4Al-3La-2Sm-0.3Mn	HPDC	266	170	11.2	[73]
Mg-4Al-3La-2Gd-0.3Mn	HPDC	284	181	14	[74]
Mg-4Al-1Ce-0.3Mn	HPDC	232	146	9	[53]
Mg-4Al-2Ce-0.3Mn	HPDC	247	148	12	
Mg-4Al-4Ce-0.3Mn	HPDC	250	157	11	
Mg-4Al-6Ce-0.3Mn	HPDC	254	161	10	
Mg-4Al-1La-0.3Mn	HPDC	236	133	12	[58]
Mg-4Al-2La-0.3Mn	HPDC	245	140	13	
Mg-4Al-4La-0.3Mn	HPDC	265	155	12	
Mg-4Al-6La-0.3Mn	HPDC	257	171	7	
Mg-4Al-1Ce/La-0.3Mn	HPDC	233	128	11	[59]
Mg-4Al-2Ce/La-0.3Mn	HPDC	240	137	11	
Mg-4Al-4Ce/La-0.3Mn	HPDC	270	160	13	
Mg-4Al-6Ce/La-0.3Mn	HPDC	261	173	8	
Mg-4Al-2RE-2Ca-0.3Mn	HPDC	234	202	4	[51]
Mg-4Al-1.5Ce-1.5La-1Si	HPDC	263	167	12	[52]
AE44	HPDC	247	147	11	[53]
Mg-4Al-4RE-0.3Mn	HPDC	284	192	11.4	[54]
AZ91-0.5RE-0.2Sr	HPDC	263	165	7.6	[60]
AZ91-1.0Ce	HPDC	248	158	6.8	[75]
AZ91-1.0Nd	HPDC	258	164	5.6	[76]
AZ91-0.5Y	HPDC	270	162	10	[37]
AZ91-0.8Pr	HPDC	228	137	6.8	[77]
AZ91-1.5Ca-1.0Y	HPDC	241	183	3.2	[38]

* HPDC: high-pressure die casting.

**Table 3:** Tensile properties of typical Mg-Zn-RE alloys at room temperature.

Alloys (wt.%)	Process	UTS (MPa)	YS (MPa)	EL (%)	Ref.
Mg-9Zn-9Y-0.6Zr	Extruded	351	245	11.0	[78]
Mg-8Zn-6Al-1Gd	Extrusion	360	273	13.5	[79]
Mg-5Zn-12Y-0.6Zr	Heat treatment	429	351	2	[78]
Mg-4.2Zn-5.7Y	Extruded	420	390	5	[88]
Mg-3.6Zn-0.6Y-0.2Ca	Extrusion	357	317	6.4	[80]
Mg-3.5Zn-9.5Y-1Mn	Extruded	421	333	5.8	[89]
Mg-3Zn-6Y-1Al	HPDC *	281	175	9.5	[18]
Mg-2.5Zn-6.8Y	Extruded	410	350	6	[90]
Mg-2.2Zn-9.5Gd-4Y-0.5Zr	Extrusion	494	425	11.2	[91]
Mg-2Zn-13Gd-4Y-0.5Zr	Extrusion	356	332	8	[92]
Mg-2Zn-11Y-5Gd-0.5Zr	Heat treatment	307	240	1.4	[93]
Mg-2Zn-14Gd-0.5Zr	Heat treatment	404	292	5.3	[94]
Mg-2Zn-9Gd-3Y-0.4Zr	Extrusion	357	242	9	[95]
Mg-2Zn-10Y	Extruded	520	-	-	[96]
Mg-2Zn-4Y-0.5Al	Extruded	416	376	11	[97]
Mg-1.5Zn-0.25Gd	Extrusion	444	408	12.5	[81]
Mg-1.2Zn-0.6Er-0.6Y-0.2Mn	Extrusion	351	320	10.0	[82]
Mg-0.5Zn-0.5Y-0.15Si	Extrusion	348	248	19	[83]

* HPDC: high-pressure die casting.

**Table 4:** Room-temperature tensile properties of Mg–RE alloys with high RE content.

Alloys (wt.%)	Process	UTS (MPa)	YS (MPa)	EL (%)	Ref.
Mg-18Gd-2Ag-0.3Zr	Heat treatment	414	293	2.2	[105]
Mg-17.4Gd-1.1Zn-0.6Zr	Heat treatment	410	313	1.9	[106]
Mg-15.6Gd-1.8Ag-0.4Zr	Heat treatment	423	328	4.9	[107]
Mg-15Gd-1Zn-0.4Zr	Extrusion	423	359	10	[108]
Mg-14.5Gd-2.3Y-1.1Zn-0.3Mn	Extrusion	520	448	3.5	[28]
Mg-14Gd-2Er-0.4Zr	Extrusion	490	481	3.2	[29]
Mg-12.6Gd-1.3Y-0.9Zn-0.5Mn	Extrusion	564	543	1.2	[109]
Mg-11.5Gd-4.5Y-1.5Zn-0.4Zr	Extrusion	453	387	8.3	[110]
Mg-11Gd-2Ag	Extrusion	393	435	5.2	[111]
Mg-10Gd-3Y-1.0Zn-0.5Zr	Extrusion	347	231	11	[23]
Mg-10Gd-3Y-0.8Al	Heat treatment	301	213	12.1	[24]
Mg-10Gd-2Y-1Zn-0.5Zr	Heat treatment	351	252	10.2	[110]
Mg-10Gd-0.4La-0.1Zn-0.4Zr	Heat treatment	397	247	5.8	[112]
Mg-10Gd-1Zn-0.5Zr	Heat treatment	303	205	6.6	[113]
Mg-9.5Gd-3.8Y-0.6Zr	Extrusion	560	518	4.8	[32]
Mg-9.2Gd-3.3Y-1.2Zn-0.9Mn	Extrusion	525	420	6.3	[33]
Mg-9Gd-4Y-0.5Zr	Heat treatment	370	277	4.5	[103]
Mg-9Gd-3Y-2Zn-0.4Zr	Extrusion	357	242	9	[97]
Mg-9Gd-3Y-0.5La-0.5Zr	Extrusion	496	480	5.8	[104]
Mg-9Gd-1Yb-0.5Zn-0.2Zr	Heat treatment	283	229	1.2	[26]
Mg-8.8Gd-3.4Y-1Zn-0.8Mn	Extrusion	415	362	8.3	[114]
Mg-8.5Gd-2.3Y-1.8Ag-0.4Zr	Heat treatment	403	268	4.9	[115]
Mg-8.3Gd-4.2Y-1.4Zn-1.1Mn	Extrusion	388	282	16.4	[116]
Mg-8.3Gd-1.1Dy-0.4Zr	Heat treatment	355	261	3.8	[27]
Mg-8.2Gd-3.8Y-1.0Zn-0.4Zr	Extrusion	434	417	12.9	[117]
Mg-8.1Gd-4Y-1Zn	Extrusion	373	303	11.0	[118]
Mg-8Gd-4Y-1Mn-0.4Sc	Extrusion	425	352	10.6	[119]
Mg-8Gd-3Yb-1.2Zn-0.5Zr	Extrusion	425	413	5.5	[120]
Mg-7Gd-3Nd-0.4Zr	Heat treatment	302	201	4.3	[112]
Mg-6Gd-3Y-0.5Zr	Heat treatment	248	173	17.5	[120]
Mg-6Gd-2Y-1Nd-1.5Ag-0.4Zn-0.4Zr	Heat treatment	220	148	5.4	[35]
Mg-4.3Gd-3.2Y-1.2Zn-0.5Zr	Heat treatment	351	303	20	[121]
Mg-19Y-6.5Ni	Heat treatment	526	460	8	[122]
Mg-7Y-4Gd-1.5Zn-0.4Zr	Heat treatment	418	320	6.2	[123]
Mg-4Y-4Sm-0.5Zr	Heat treatment	348	217	6.9	[124]
Mg-6Er-3Y-1.5Zn-0.4 Mn	Extrusion	354	316	8.1	[125]

**Table 5:** Room-temperature tensile properties of Mg–RE alloys with low RE content.

Alloys (wt.%)	Process	UTS (MPa)	YS (MPa)	EL (%)	Ref.
Mg-3Nd-4.5Gd-0.2Zn-0.5Zr	Heat treatment	343	200	5.4	[18]
Mg-3Nd-3Gd-0.5Zn-0.5Zr	Heat treatment	301	179	5.3	[19]
Mg-3Nd-2.6Gd-0.2Zn-0.5Zr	Heat treatment	303	220	4.1	[20]
Mg-3Nd-1Gd-0.3Zn-0.4Zr	Heat treatment	230	173	6.5	[4]
Mg-3Nd-0.4Zn-0.4Zr	Heat treatment	198	116	14.0	[21]
Mg-3Nd-0.2Zn-0.4Zr	Heat treatment	296	266	4.9	[22]
Mg-4Y-2.4Nd-0.2Zn-0.4Zr	Heat treatment	339	268	4	[140]
Mg-3.5Y-2Nd-1.3Gd-0.4Zr	Heat treatment	345	196	7	[34]
Mg-2Y-0.5Zn-0.5Ni	Extrusion	389	336	12.6	[30]
Mg-4Sm-1Nd-0.6Zn-0.4Zr	Heat treatment	190	138	5.5	[112]
Mg-4Sm-0.6Zn-0.4Zr	Heat treatment	462	458	4.8	[140]
Mg-3.5Sm-2Yb-0.6Zn-0.4Zr	Extrusion	451	449	4.9	[120]
Mg-3.5Sm-0.6Zn-0.5Zr	Extrusion	381	363	9.0	[31]

**Table 6:** Room-temperature tensile properties of multicomponent magnesium alloys containing RE elements.

Alloys (wt.%)	Process	UTS (MPa)	YS (MPa)	EL (%)	Ref.
Mg-0.2Ca-3.6Zn-0.6Y	Extrusion	357	317	6.4	[80]
Mg-0.4Ca-1.2Al-0.2Ce	Extrusion	358	350	12.1	[48]
Mg-0.8Ca-0.4Mn-0.2Ce	Extrusion	430	428	2	[144]
Mg-1Ca-4Al-3La-0.3Mn	HPDC	274	220	6.3	[68]
Mg-1Ca-10Gd-1Al	Heat treatment	211	163	11.6	[145]
AZ91-1.5Ca-1.0Y	HPDC	241	183	3.2	[38]
Mg-2Ca -4Al-2RE-0.3Mn	HPDC	234	202	4	[51]
Mg-0.15Si-0.5Zn-0.5Y	Extrusion	348	248	19	[83]
Mg-1Si-4Al-1.5Ce-1.5La	HPDC	263	167	12	[52]
Mg-0.3Mn-2Er	Extrusion	201	134	35.3	[146]
Mg-0.9Mn-9.2Gd-3.3Y-1.2Zn	Extrusion	525	420	6.3	[33]
Mg-1Mn-8Gd-4Y-0.4Sc	Extrusion	425	352	10.6	[119]
Mg-1.1Mn-8.3Gd-4.2Y-1.4Zn	Extrusion	388	282	16.4	[116]
Mg-1.5Ag-6Gd-2Y-1Nd-0.4Zn-0.4Zr	Heat treatment	220	148	5.4	[35]
Mg-1.8Ag-15.6Gd-0.4Zr	Heat treatment	423	328	4.9	[107]
Mg-1.8Ag-8.5Gd-2.3Y-0.4Zr	Heat treatment	403	268	4.9	[115]
Mg-2Ag-18Gd-0.3Zr	Heat treatment	414	293	2.2	[105]
Mg-2Ag-11Gd	Extrusion	393	435	5.2	[111]
Mg-2.0Ag-7.8Gd-2.7Y-0.4Zr	Heat treatment	410.7	273.1	4.85	[147]
AZ91-0.2Sr-0.5RE	HPDC	263	165	7.6	[60]
Mg-0.5Ni-2Y-0.5Zn	Extrusion	389	336	12.6	[30]

## Data Availability

No new data were created or analyzed in this study. Data sharing is not applicable to this article.
